# MAIGRET: a CRISPR-based immunoassay that employs antibody-induced cell-free transcription of CRISPR guide RNA strands

**DOI:** 10.1093/nar/gkaf238

**Published:** 2025-03-28

**Authors:** Francesca Miceli, Sara Bracaglia, Daniela Sorrentino, Alessandro Porchetta, Simona Ranallo, Francesco Ricci

**Affiliations:** Department of Chemical Sciences and Technologies, University of Rome, Tor Vergata, Via della Ricerca Scientifica 1, 00133 Rome, Italy; Department of Chemical Sciences and Technologies, University of Rome, Tor Vergata, Via della Ricerca Scientifica 1, 00133 Rome, Italy; Department of Chemical Sciences and Technologies, University of Rome, Tor Vergata, Via della Ricerca Scientifica 1, 00133 Rome, Italy; Department of Mechanical and Aerospace Engineering and of Bioengineering, University of California at Los Angeles, 420 Westwood Plaza, Los Angeles, CA 90095, United States; Department of Chemical Sciences and Technologies, University of Rome, Tor Vergata, Via della Ricerca Scientifica 1, 00133 Rome, Italy; Istituto Nazionale Biostrutture e Biosistemi, INBB, Via dei Carpegna, 00165 Rome, Italy; Department of Chemical Sciences and Technologies, University of Rome, Tor Vergata, Via della Ricerca Scientifica 1, 00133 Rome, Italy; Istituto Nazionale Biostrutture e Biosistemi, INBB, Via dei Carpegna, 00165 Rome, Italy; Department of Chemical Sciences and Technologies, University of Rome, Tor Vergata, Via della Ricerca Scientifica 1, 00133 Rome, Italy; Istituto Nazionale Biostrutture e Biosistemi, INBB, Via dei Carpegna, 00165 Rome, Italy

## Abstract

Here we report on the development of a CRISPR-based assay for the sensitive and specific detection of antibodies and antigens directly in complex sample matrices. The assay, called Molecular Assay based on antibody-Induced Guide-RNA Enzymatic Transcription (MAIGRET), is based on the use of a responsive synthetic DNA template that triggers the cell-free *in vitro* transcription of a guide RNA strand upon recognition of a specific target antibody. Such transcribed guide RNA activates the DNA collateral activity of the Cas12a enzyme, leading to the downstream cleavage of a fluorophore/quencher-labeled reporter and thus resulting in an increase in the measured fluorescence signal. We have used MAIGRET for the detection of six different antibodies with high sensitivity (detection limit in the picomolar range) and specificity (no signal in the presence of non-target antibodies). MAIGRET can also be adapted to a competitive approach for the detection of specific antigens. With MAIGRET, we significantly expand the scope and applicability of CRISPR-based sensing approaches to potentially enable the measurement of any molecular target for which an antibody is available.

## Graphical Abstract

**Graphical Abstract:**
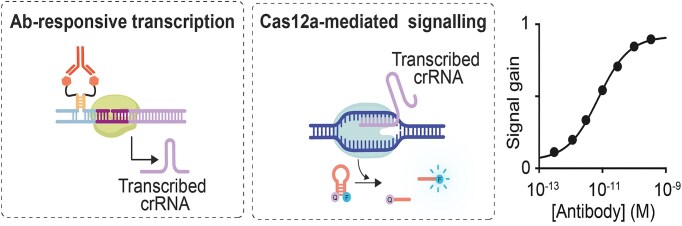

## Introduction

In addition to their clinical applications in cell and gene therapies, CRISPR-Cas enzymes are also gaining increasing importance as versatile tools for sensor applications [1–6]. This is mainly due to the ability of type V and VI CRISPR-Cas systems to combine target recognition, signal transduction and amplification. More specifically, the discovery of the non-specific collateral cleavage (*trans*-cleavage) of Cas12 and Cas13 systems has led to the development of many sensing platforms for the ultrasensitive detection of specific nucleic acid sequences [7–10]. Such CRISPR-based sensors employ nucleic acid reporters that, in response to the target recognition, are cleaved by the collateral activity to produce a measurable output [11, 12]. For example, by combining the RNA collateral effect of Cas13a with a pre-amplification step, Zhang, Collins and colleagues have developed a CRISPR-based diagnostic platform called SHERLOCK, that enables the detection of specific DNA sequences from viruses and bacterial species with attomolar sensitivity and single-base mismatch specificity [13, 14]. Recently, an improved version of SHERLOCK has been proposed that allows multiplexing and adaptation to lateral flow reading [15, 16]. Doudna's group has also demonstrated the use of an alternative CRISPR system, called DETECTR, as a detection tool for DNA-specific sequences [17]. In DETECTR, the DNA target sequence is first amplified by Recombinase Polymerase Amplification (RPA) and the amplified sequence is recognized by Cas12, which, unlike Cas13, recognizes DNA sequences and therefore does not require the DNA-to-RNA transcription step used in SHERLOCK. Considering the above advantages of CRISPR-based sensing tools, it would be extremely important to adapt similar approaches to the detection of non-nucleic acid targets, a goal that has proven difficult to achieve.

The main challenge in the development of CRISPR sensors for non-nucleic acid targets is the need to optimize a nucleic acid-mediated approach to convert target recognition into the collateral cleavage activity of Cas effectors. This is one of the main reasons why, despite the significant advances made in the last years, only few examples of CRISPR-based sensors have been demonstrated for the detection of non-nucleic acid targets. In this context, the DETECTR technology described above has recently been adapted to the detection of various small molecules, including uric acid, adenosine 5′-triphosphate and metal ions by using bacterial allosteric transcription factors (TFs) or aptamers as recognition units [18–21]. Recently, CRISPR-based sensing platforms that enable the detection of clinically relevant proteins and antibodies have been also described [22, 23]. In one example (named UCAD) CRISPR cleavage activity was coupled with recombinase polymerase amplification (RPA) and with antibody-conjugated DNA strands to demonstrate sensitive detection of COVID protein targets with high specificity [22]. In another example, DNA-based toehold switches were rationally designed to control Cas12a activity in response to various molecular inputs including small molecules and antibodies by taking advantage of PAM accessibility [23]. The above examples clearly demonstrate the adaptability of CRISPR-based approaches for sensing applications. However, they also highlight the urgent need for new strategies to develop more versatile and sensitive tools that can detect a wide range of targets and be applied for diagnostic and sensing applications.

Motivated by the above considerations, here we demonstrate a two-step CRISPR-based immunoassay, which, in analogy with the nucleic-acid detection system SHERLOCK, we have named MAIGRET (Molecular Assay based on antibody-Induced Guide-RNA Enzymatic Transcription). MAIGRET enables versatile detection of specific antibodies and other proteins with high sensitivity by combining the advantageous properties of CRISPR-based sensors with those of cell-free transcription systems. Cell-free transcription systems consist of nucleotides, polymerase enzymes and synthetic templates programmed to achieve *in vitro* transcription of an RNA signalling strand in an input-responsive manner [24–26]. Cell-free transcription assays have been developed so far for the detection of viral RNA and small molecules [27, 28]. More recently, we have developed cell-free transcription-based sensors for antibody detection, in which the binding of a specific antibody to antigen-conjugated nucleic acid strands [29] triggers the *in vitro* transcription of a signalling RNA strand [30, 31]. However, despite the enzymatic transcription reaction, the above system generally lacks the sensitivity to compete with other enzyme-based immunoassays such as ELISA. With MAIGRET, we overcome this limitation by combining cell-free transcription systems with CRISPR-based signal amplification.

## Materials and methods

Reagent-grade chemicals 4- (2-hydroxyethyl)-1-piperazineethanesulfonic acid (HEPES), magnesium chloride (MgCl_2_), tris-hydrochloride (Tris-HCl), sodium chloride (NaCl), potassium chloride (KCl) and bovine serum albumin (BSA) were purchased from Sigma-Aldrich (St Louis, Missouri) and used without further purifications. Sheep polyclonal Anti-Dig antibodies were purchased from Roche Diagnostic Corporation, (Germany), mouse monoclonal Anti-DNP antibodies and rabbit polyclonal Anti-mouse IgG antibodies were purchased from Sigma-Aldrich, (USA), rat monoclonal Anti-FLAG antibodies were purchased from Novus Biologicals (UK), murine monoclonal Anti-HIV antibodies were purchased from Zeptometrix Corporation (USA). Cetuximab, monoclonal Anti-MUC antibodies, bi-specific antibodies and EGFR protein were kindly provided by Merck (Darmstadt, Germany). Human-Mucine 1 protein was purchased from ACRO Biosystems (USA). HIV-1 p17 peptide was purchased from biorbyt (USA). All the antibodies were aliquoted and stored at 4°C for immediate use or at − 20°C for long- term storage. LbaCas12a from *Lachnospiraceae bacterium ND2006* is expressed as a N-terminal 6XHis-tagged fusion in *E. coli*. It was purchased from New England Biolabs (USA). It was aliquoted and stored at -20°C for long term storage. LwaCas13a from *Leptotrichia wadei* is a recombinant full-length Cas13a protein with a His tag expressed in *E. coli*. It was purchased from SignalChem Lifesciences Corporation (Canada). It was aliquoted and stored at -70°C for long term storage.

### Oligonucleotides

HPLC-purified oligonucleotides were purchased from Biomers (Germany), Biosearch Technologies (Risskov, Denmark) and Metabion International AG (Planegg, Germany) as lyophilized powder after HPLC purification. PNA/Peptide chimera probes were purchased from Panagene (South Korea). All sequences were designed using Nupack or IDT oligoanalyzer tools and are reported in the Supplementary Data document (Supplementary Table S1-Supplementary Table S6) [32, 33]. All oligonucleotides were dissolved in DEPC water at a concentration of 100 μM and stored at -20°C.

Conjugation of DBCO modified DNA strands to the protein (EGFR) was performed using ProFire® (Dynamic Biosensors, Germany) and amine coupling kit following the manufactures instructions. For that, 4 nmol of DNA was conjugated with 400 μg of EGFR overnight. The crosslinking products were separated by ion exchange chromatography using the ProFire® equipment and after the data analysis with the equipment software, the fractions that correspond to 1:1 binding ratio were collected and stored at -20°C.

### Cell-free transcription reactions

All transcription reactions were performed using a ThermoFisher TranscriptAid T7 high yield transcription kit (ref. K0441) following the recommended manufacturer protocols. More specifically, we have prepared the 10 μL solution of the commercial transcription kit so that they contain the inactive template (3 nM for Cas12a system and 0.3 nM for Cas13a system), the antibody-responsive module (30 nM for Cas12a system and 3 nM for Cas13a system), and the antibody (when indicated). The transcription reaction was allowed to proceed for 120 min at 37°C after T7 polymerase addition.

### Cleavage reactions

An aliquot of the transcription reaction solution (2 μL) was transferred to a 8 μL solution of 10 mM Tris-HCl, 50 mM NaCl, 10 mM MgCl_2_, pH 7.8, containing the relevant Cas enzyme (Cas12a 200 nM or Cas13a 30 nM) and the DNA activator (10 nM) for Cas12a system or the RNA activator (2 nM) for Cas13a system. The cleavage reaction was allowed to proceed for 30 min at 37°C after the Cas enzyme addition.

### Fluorescence measurements

For reactions using Cas12a system, an aliquot (2 μL) of the cleavage reaction solution (see above) was transferred to an 18 μL solution of 10 mM Tris-HCl, 50 mM NaCl, 10 mM MgCl_2_, pH 7.8, containing 100 nM of the DNA reporter specific for Cas12a system.

For reactions using Cas13a system, an aliquot of the cleavage reaction solution (2 μL) was transferred to a 18 μL solution of 80 mM HEPES, 200 mM KCl, 20 mM MgCl_2_, 0.4 mg/mL BSA, pH 7, containing 30 nM of the RNA reporter specific for Cas13a system.

Fluorescence measurements were carried out on a Tecan F200pro plate reader using the top reading mode with black 384-well plates and a 20 μL final volume. Equilibrium fluorescence measurements were obtained respectively with excitation at 485 (± 20) nm and emission at 535 (± 25) nm (for the Cas12a reporter labeled with 6-FAM (6- Carboxyfluorescein)) or with excitation at 540 (± 25) nm and emission at 595 (± 35) nm (for the Cas13a reporter labeled with Cy3 (Cyanine-3)).

Curve fitting at different concentrations of the target antibody was obtained using Prism- GraphPad software and its built-in Hill function with the following Lavenberg–Marquardt iteration algorithm:


\begin{eqnarray*}
{{{\mathrm{F}}}_{\left[ {{\mathrm{Antibody}}} \right]}} = {\mathrm{\ }}{{{\mathrm{F}}}_{{\mathrm{min}}}} - \left( {{{{\mathrm{F}}}_{{\mathrm{max}}}} - {\mathrm{\ }}{{{\mathrm{F}}}_{{\mathrm{min}}}}} \right)\frac{{{{{\left[ {{\mathrm{Antibody}}} \right]}}^{{{{\mathrm{n}}}_{\mathrm{H}}}}}}}{{{\mathrm{K}}_{\mathrm{D}}^{{{{\mathrm{n}}}^{\mathrm{H}}}} + {\mathrm{\ }}{{{\left[ {{\mathrm{Antibody}}} \right]}}^{{{{\mathrm{n}}}_{\mathrm{H}}}}}}}\end{eqnarray*}

where, F_min_ and F_max_ are the minimum and maximum fluorescence values respectively, K_D_ is the equilibrium antibody concentration at half-maximum signal, n_H_ is the Hill coefficient, and [Antibody] is the concentration of the specific target antibody added.

### Cetuximab determination with ELISA

Cetuximab ELISA kit (Erbitux®) purchased from AssayGenie was used to detect Cetuximab. Spiked serum samples, buffer and reagents were prepared following the manufacturer's instructions involving the following steps. First, standards and spiked serum samples were diluted 100 times with assay buffer, and 100 μL were added to different wells of the microplate already containing 100 μL of assay buffer and incubated for 30 min. Next, the liquid was removed, and the wells were washed 3 times with 300 μL of washing buffer. 100 μL of the HRP-conjugate reporter was then added and incubated for 30 min. Next, the wells were washed 3 times and 100 μL of TMB substrate solution was added and incubated for 10 min in the dark. Finally, 100 μL of a stop solution was added, and the absorbance was measured at 450 nm with MultiSkan Sky (ThermoFisher).

## Results and discussion

**Figure 1.**
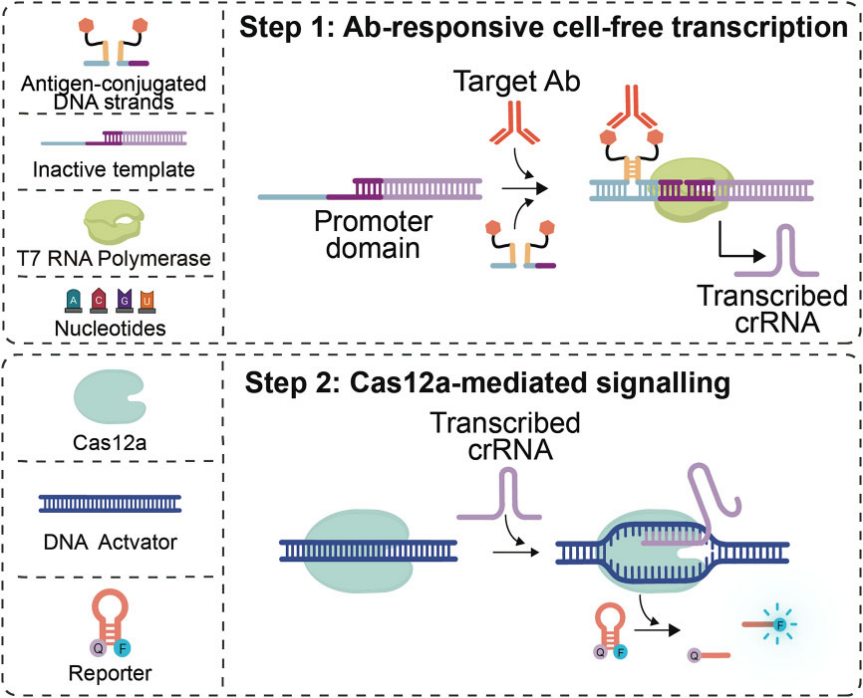
MAIGRET is a two-step CRISPR-based assay for the sensitive and specific detection of antibodies and antigens. The first step of MAIGRET involves the use of antigen-conjugated DNA strands that can hybridize to an incomplete synthetic DNA template only upon recognition of a specific target antibody and induce cell-free transcription of a CRISPR RNA (crRNA) guide strand. In the second step, the transcribed crRNA triggers the collateral cleavage of an optically-labelled hairpin DNA reporter by Cas12a enzyme leading to an increase in fluorescence signal.

MAIGRET comprises two separate reaction steps. In the first step of the assay (antibody-responsive cell-free transcription) the binding of a target antibody to a pair of antigen-conjugated DNA strands induces the formation of a bimolecular complex by co-localization, which can hybridize to the single-stranded part of an incomplete inactive synthetic template encoding the CRISPR RNA (crRNA) guide strand specific of Cas12a enzyme. Only when such a bimolecular complex is formed the synthetic template is activated and cell-free transcription of the crRNA strand can begin (Fig. 1, top). In the second step (Cas12a-mediated signalling), an aliquot of the reaction from the first step is transferred to a solution containing Cas12a, its double-stranded DNA activator and a DNA hairpin reporter labelled with a fluorophore/quencher pair (stem-loop DNA reporters have been recently demonstrated to improve signalling of Cas12a *trans*-cleavage activity) [34]. The presence of the crRNA transcribed in step 1 induces the DNA collateral activity of Cas12a, leading to cleavage of the reporter and the resulting increase in the measured fluorescence signal (Fig. 1, bottom).

**Figure 2.**
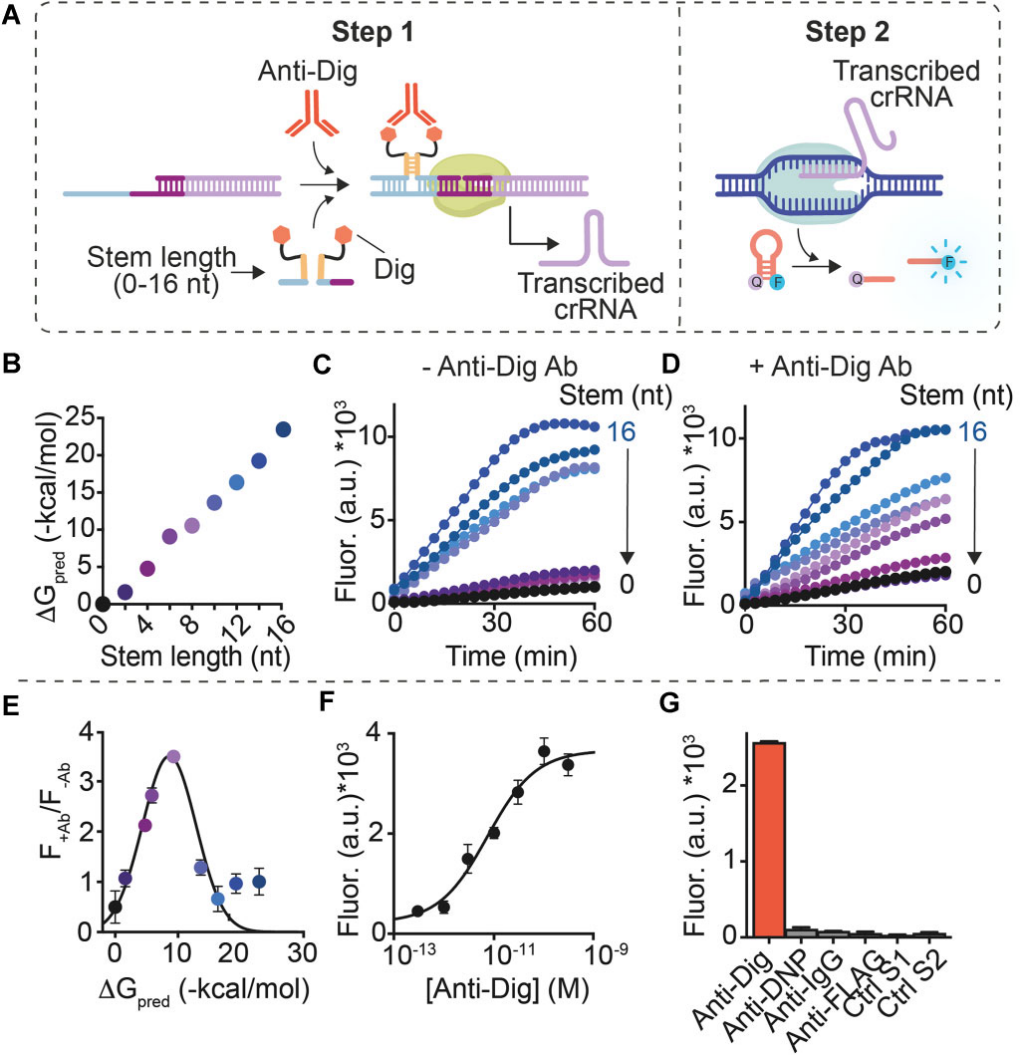
(**A**) Simplified scheme of MAIGRET for the detection of Anti-Dig antibodies using Dig-conjugated DNA strands. (**B**) Predicted free hybridization energy vs length of the complementary portions of the Dig-conjugated DNA strands used here. (**C** and **D**) Signals obtained in the absence and presence (300 pM) of Anti-Dig antibodies with each Dig-conjugated DNA strand pair. (**E**) Plot of the ratio between the signal obtained in the presence (F_+Ab_) and absence (F_-Ab_) of Anti-Dig antibodies (300 pM) versus the predicted free hybridization energy of the Dig-conjugated DNA strand pairs. (**F**) Binding curve at increasing Anti-Dig concentrations using the Dig-conjugated DNA strands with 8-nt complementary portion. (**G**) Fluorescence signals obtained in the presence of saturating concentrations (300 pM) of Anti-Dig antibody and non-specific antibodies and in control experiments (Ctrl S1 and Ctrl S2). The experiments were carried out as follows. First, transcription was performed at 37°C in a 10 μL buffer solution containing T7-RNAP (10 U/μL), the required nucleotides (each at 10 mM), the inactive template (3 nM), the antigen-conjugated strands (each at 30 nM) and, where indicated, the Anti-Dig antibody or non-specific antibody. The transcription reaction was allowed to proceed for 120 min and then an aliquot (2 μL) was transferred to a 18 μL a buffer solution (10 mM Tris-HCl, 50 mM NaCl, 10 mM MgCl_2_, pH 7.8) containing Cas12a (20 nM), the DNA activator (1 nM), and the DNA reporter (100 nM). In panels E-G the end-point fluorescence signal was measured after 60 min. For these and the following experiments, the values represent averages of three separate measurements and the error bars reflect the standard deviations.

To optimize MAIGRET, it is important to find the optimal thermodynamic conditions under which *in vitro* transcription of crRNA occurs exclusively after binding of the antibody to the two antigen-conjugated strands. To this end, we have first designed a dsDNA template in which the promoter binding domain responsible for recognition by T7 RNA polymerase is incomplete and thus inactive. Such template can be efficiently activated by a 21-nt DNA strand that completes the promoter binding domain and induces transcription of the crRNA strand (Supplementary Fig. S1). We have then selected a simple antigen–antibody pair: we used digoxigenin (Dig), a small molecule (MW = 390.51 g/mol) that can be easily conjugated to DNA strands (by EDC/NHS reaction), as antigen and Anti-Dig-IgG antibody as the target. Finally, we designed and synthesized a series of Dig-conjugated DNA strand pairs in which each pair has a complementary stem-forming domain with variable length (from 0 to 16 nucleotides) and thus stability (estimated free energy values from 0 to −23 kcal/mol) (Fig. 2A and B). The hybridization between these two Dig-conjugated strands would form a bimolecular complex that is able to efficiently bind and activate the incomplete template (of note, the presence of the stem does not affect transcription as demonstrated with a control strand containing a stem loop portion, Supplementary Fig. S2).

With each of the Dig-conjugated DNA pairs described above, we performed cell-free transcription reactions (step 1) in the absence and presence of Anti-Dig antibodies (300 pM) and transferred an aliquot of the reaction solution to an Eppendorf tube containing Cas12a (20 nM), the DNA activator (1 nM), and the DNA hairpin reporter (100 nM) (step 2) (Fig. 2C and D). We note here that this optimization step was performed at 37°C as this represents the optimal temperature for both T7 polymerase and Cas12a enzyme. In the absence of Anti-Dig antibodies, we found that Dig-conjugated strands with complementary portions shorter than eight bases resulted in low reporter signals, likely due to the intrinsic instability of the duplex formed between the strands, which ultimately prevents efficient activation of the synthetic template (Fig. 2C). This hypothesis is confirmed by the fact that Dig-conjugated strands with longer stem-forming domains (>8 nucleotides) provide gradually increasing signals. As expected, the same experiments performed in the presence of Anti-Dig antibodies show signals that are already measurable with shorter stem-forming domains (i.e. 6 nucleotides), supporting the role of co-localization induced by the target antibody (Fig. 2D). A stem-forming domain of 8 bases provides the largest signal change between the absence and presence of the Anti-Dig antibody and was therefore selected for further experiments (Fig. 2E). Using these antigen-conjugated strands, we tested increasing concentrations of Anti-Dig antibody (from 1 × 10^–12^ M to 300 × 10^–12^ M) and obtained a dose-response curve with an limit of detection (LOD, defined here as the anti-Dig concentration that gives a signal three times greater than the standard deviation of a blank solution) of 2 × 10^–13^ M with a dynamic range (defined here as the anti-Dig concentration range in which we obtain signals between 10% and 90% of the maximum signal) between 8 × 10^–13^ and 4 × 10^–11^ and an average RSD% of 4% (Fig. 2F). MAIGRET is highly specific: we observe only minimal leakage at saturating concentrations of non-specific antibodies and in other control experiments where only one of the two antigen-conjugated strands is used (Ctrl S1 and Ctrl S2, Fig. 2G).

**Figure 3.**
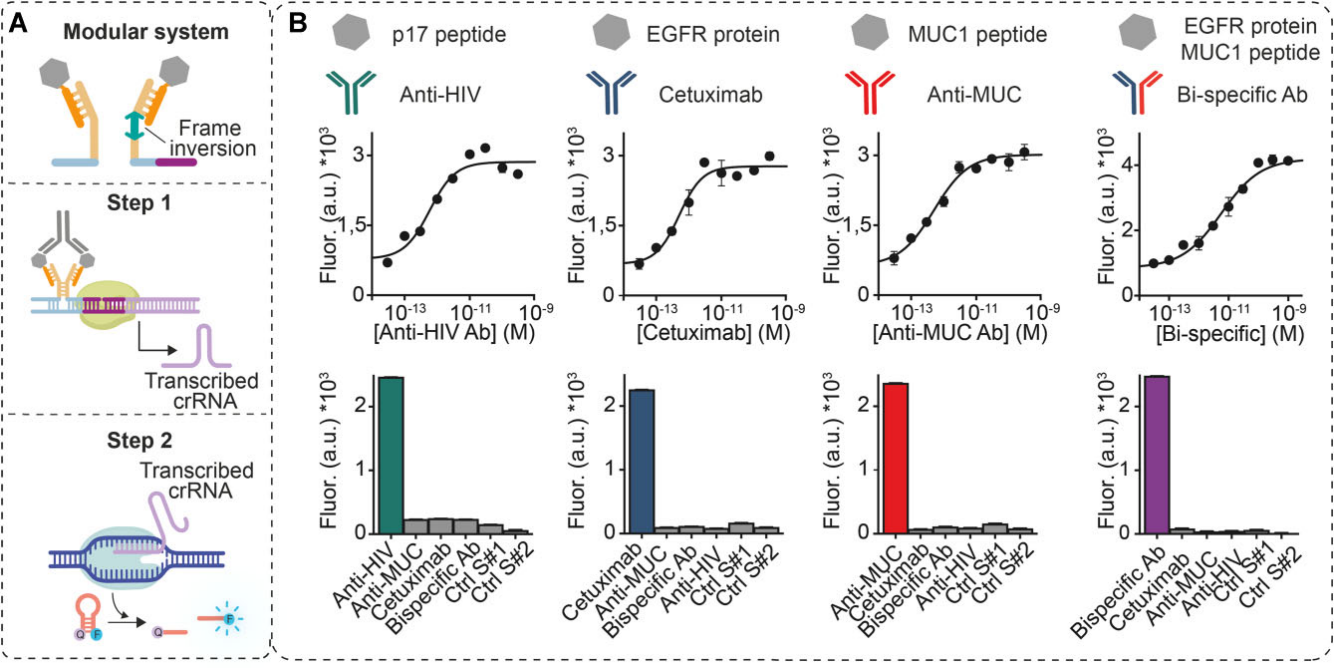
(**A**) Simplified scheme of the modular version of MAIGRET in which antigen-conjugated DNA strands (top) hybridize to a pre-optimized pair of DNA strands able to activate the synthetic template. A frame inversion in one of the template-activating strands allows the same antigen-conjugated strand to be used in the assay. (**B**) Each panel shows the antigen used (p17 peptide, EGFR, MUC1, EGFR and MUC1), the corresponding antibody target detected by each modular version of MAIGRET, the binding curve at increasing target concentrations and the fluorescence signals obtained in the presence of saturating concentrations (300 pM) of the target antibody and non-specific antibodies and in control experiments (Ctrl S1 and Ctrl S2). The experimental procedure used here is the same as described in Fig. 2, except that the transcription step (step 1) of the assay was performed in a solution prepared by mixing the buffer solution and bovine serum in a 1:1 ratio. The concentration of the antigen-conjugated strand used was 60 nM, while the concentration of each template-activating strand was 30 nM.

MAIGRET can also be generalized to the detection of potentially any target antibody by simply changing the recognition elements (i.e. the antigens) conjugated to the DNA strand pairs. To facilitate the application of the approach to the use of peptide- or protein-based antigens, we developed a modular version in which the antigen-conjugated strands hybridize to two unmodified DNA strands responsible for stem formation and hybridization to the incomplete synthetic template (Fig. 3A). In this modular version, one of the two stem-forming DNA strands has a frame inversion so that a single antigen-conjugated DNA strand can be used to hybridize to both stem-forming strands, minimizing the overall cost of the assay (see scheme Fig. 3A). To demonstrate the applicability of our approach, we tested this modular version of MAIGRET for the detection of three different monoclonal antibodies in 50% bovine blood serum, a safe and convenient substitute for human samples. Specifically, we used three different recognition elements: a peptide with 13 residues excised from the HIV protein p17, the EGFR protein and a peptide with 15 residues excised from the Muc1 protein. We used these systems to detect Anti-HIV antibodies, Cetuximab and Anti-MUC antibodies, respectively (Fig. 3B). By using two strands of DNA conjugated to EGFR and MUC1 peptide, respectively, we were also able to demonstrate the detection of a bispecific antibody re-engineered to contain one binding site for the EGFR protein and the other for the Muc1 protein (Fig. 3B, right panel). All these modular versions of MAIGRET showed sensitivities (Anti-HIV K_1/2_ = 6 ± 1 × 10^–13^ M; Cetuximab K_1/2_ = 5 ± 1 × 10^–13^ M; Anti-MUC K_1/2_ = 8 ± 3 × 10^–13^ M; Bispecific Ab K_1/2_ = 5.5 ± 0.5 × 10^–12^ M) and specificity (no significant measurable signal in presence of non-specific targets) comparable to their non-modular counterpart with LOD values in the femtomolar range (i.e. LOD Anti-HIV = 2 × 10^–14^ M or 20 fM; LOD Cetuximab = 9 × 10^–14^ M or 90 fM; LOD Anti-MUC = 3 × 10^–14^ M or 30 fM; LOD Bispecific Ab = 7 × 10^–14^ M or 70 fM) and average RSD% < 7%.

We then proceeded to compare the analytical performance of MAIGRET with that of a commercial ELISA kit. To do this, we employed the MAIGRET assay for the detection of Cetuximab. Overall, MAIGRET offers a better sensitivity (LOD of ELISA kit = 8 × 10^–11^ M versus LOD of MAIGRET = 9 × 10^–14^ M) with fewer reaction steps (Supplementary Figs S3 and S4). The only downside of MAIGRET is represented by a longer assay time (4.5 h vs 1.5 h) that should be shortened in the future for better applicability of the approach.

We also directly compared MAIGRET with the only approach so far described based on CRISPR-Cas for the detection of proteins (i.e. UCAD^22^) (Supplementary Fig. S5). Thanks to the additional amplification step (RPA + CRISPR-Cas) UCAD allows an overall better sensitivity than MAIGRET (aM versus fM). Compared to MAIGRET, UCAD has also been demonstrated in different complex samples (i.e. serum, undiluted human serum samples). Both approaches display a good specificity and good adaptability to detect different targets.

**Figure 4.**
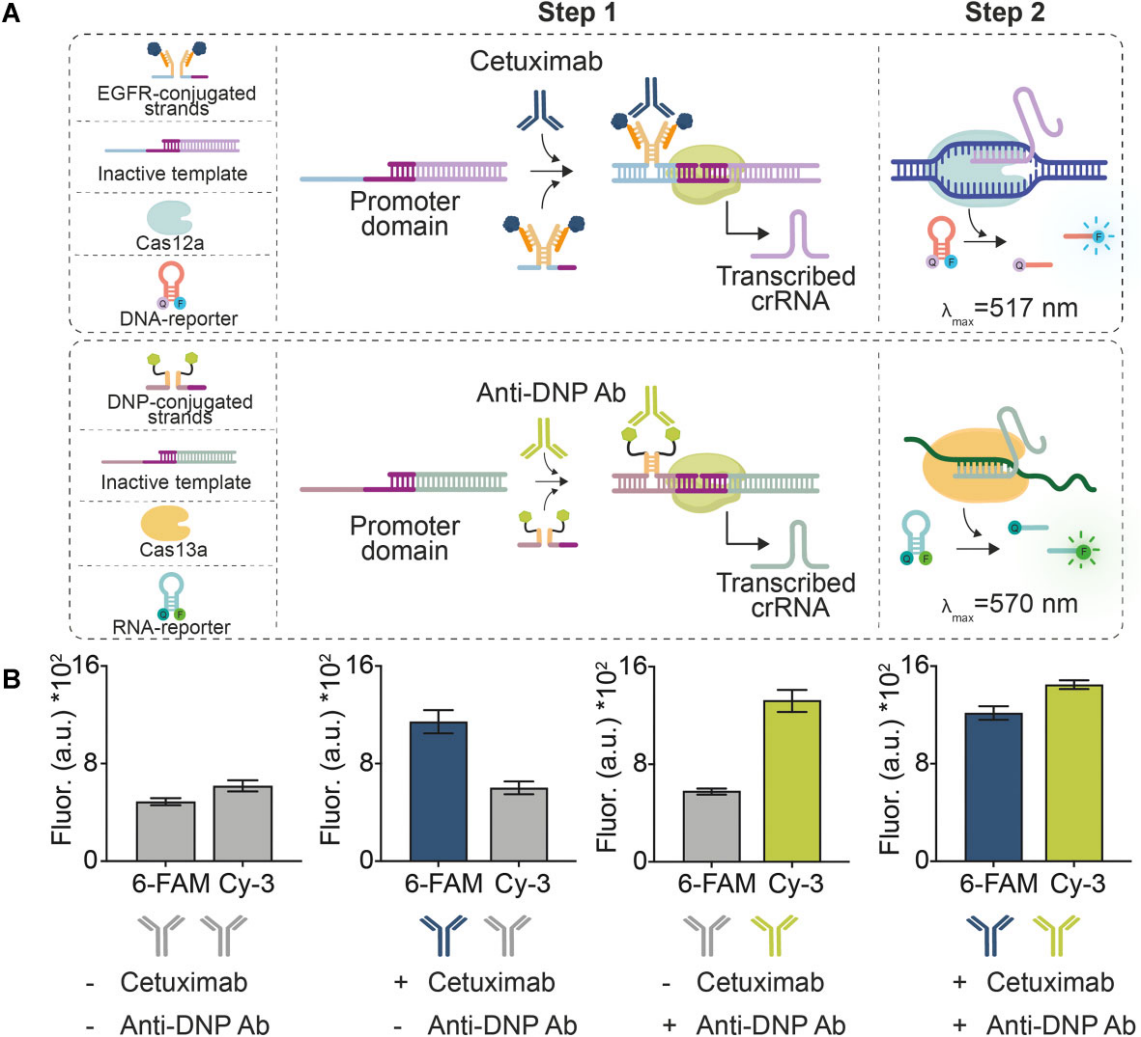
(**A**) Simultaneous detection of Anti-EGFR (Cetuximab) and Anti-DNP antibodies using MAIGRET. Each system is designed to respond to a specific antibody and transcribe a different guide RNA sequence that activates either Cas12a or Cas13a enzyme. Once activated, each Cas protein can cleave its specific reporter providing an unambiguous signal. (**B**) Fluorescence signals of the two reporters after adding different combinations of the two target antibodies. The experiments were conducted at 37°C in a 20 μL solution containing T7-RNAP (10 U/μL) and the required nucleotides (each at 10 mM) supplemented with the incomplete templates (Cas12a-based system at 3 nM, Cas13a-based system at 300 pM), unmodified DNA strand (30 nM), the antigen-conjugated strands (Cas12a-based system at 30 nM each, Cas13a-based system at 3 nM each) and the target antibodies as indicated. The transcription reaction was allowed to proceed for 120 min and then an aliquot was transferred to 18 μL of 10 mM Tris-HCl, 50 mM NaCl, 10 mM MgCl_2_, pH 7.8 solution containing Cas12a (20 nM), Cas13a (3 nM), the DNA activator (1 nM), the RNA activator (200 pM), a DNA reporter (100 nM) and an RNA reporter (30 nM).

The availability of different Cas enzymes provides an easy route towards using MAIGRET for the simultaneous measurements of different targets. To demonstrate this, we employed Cas13a RNA collateral activity in step 2 of MAIGRET. Cas13a, unlike Cas12a, preferentially cleaves RNA instead of DNA and so in this case we employed a ssRNA fluorophore/quencher reporter. We also re-engineered the antibody-responsive synthetic template and the antigen-conjugated DNA strands so that transcription of the crRNA strand specific for Cas13a could be achieved (Supplementary Fig. S6). With this new version of MAIGRET we used dinitrophenol (DNP) and Anti-DNP as antigen and target antibody respectively, measuring Anti-DNP antibodies reaching sensitivities (K_1/2_ = 16 ± 1 × 10^–12^ M, LOD = 4 × 10^–12^ M) and reproducibility (RSD% = 5%) comparable to those obtained using Cas12a enzyme as reporter enzyme in 50% bovine blood serum (Supplementary Fig. S7). Of note, because the fluorophore of the Cas13a reporter is different to that used with Cas12a and because all DNA strands have been designed to be orthogonal to the Cas12a system, it is possible to use both systems in the same solution without significant cross-talk and achieve simultaneous detection of two different antibodies. We demonstrated this using the Cas13a-based MAIGRET system for Anti-DNP detection and the Cas12a-based MAIGRET system for Cetuximab detection. By adding the relevant reagents in the same solution in the presence of various combinations of the two target antibodies (Fig. 4A), we observed measurable fluorescence only in the presence of the specific antibody (Fig. 4B). We note, however, that in this experiment, the background signals in both systems are higher than expected probably due to non-specific cleavage of the reporters from the enzymes.

**Figure 5.**
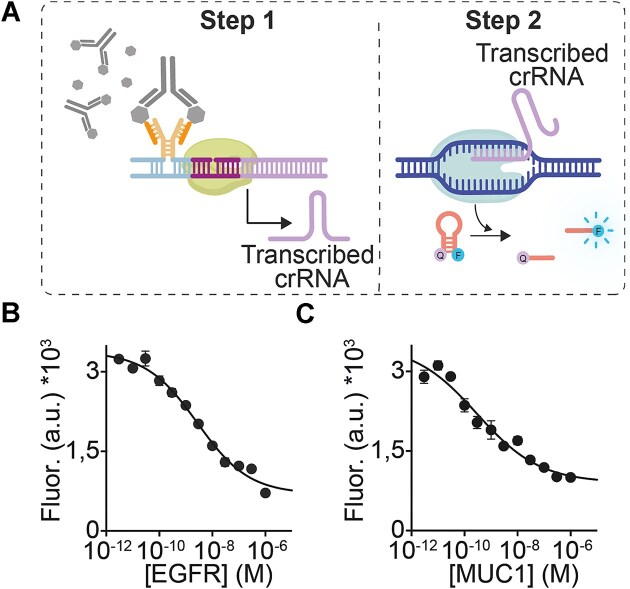
(**A**) General scheme of the competitive format of MAIGRET. (**B** and **C**) Binding curves at increasing EGFR and MUC1 concentrations using the modular version of MAIGRET described in Fig. 3. The experiments here were performed as described in Figs. 3 (also in this case in 50% serum) with the exception that the solution of the transcription step (step 1) of the assay was supplemented with a fixed concentration (3 nM) of Anti-EGFR (Cetuximab) or Anti-MUC antibody.

MAIGRET can also be adapted to a competitive assay to allow the measurement of potentially any target that can be recognized by an antibody. In this case, we used the Cas12a-based MAIGRET systems characterized above, which respond to Anti-MUC, Cetuximab and HIV antibodies. These antibodies target the cancer-related proteins MUC1 and EGFR and the HIV p17 peptide, respectively. During step 1, we have therefore added the antibody at a fixed concentration in solution and different concentrations of the free target proteins so that they compete with the antigen-conjugated DNA strands for antibody binding (Fig. 5A). The signal generated during step 2 Cas-mediated cleavage of the reporter strand is thus inversely proportional to the concentration of free target in the solution. The results we have obtained with such competitive format show that MAIGRET can be used to detect EGFR, MUC1 and HIV p17 peptide at low nanomolar concentrations in 50% serum (Fig. 5B and C, Supplementary Fig. S8).

## Conclusions

Here we have presented an ultrasensitive two-step immunoassay, called MAIGRET, for the detection of specific antibodies and antigens that combines CRISPR technology and cell-free transcription systems. The approach employs programmable synthetic DNA templates that induce cell-free *in vitro* transcription of a guide RNA strand upon recognition of a specific target antibody. The guide RNA then activates *trans*-cleavage of a fluorophore/quencher labelled reporter by the Cas enzyme leading to a measurable fluorescence signal. The dual amplification on which MAIGRET is based (i.e. RNA transcription and Cas enzyme amplification) and its modularity enable low pM detection limits directly in complex sample matrices. MAIGRET is also extremely versatile. By simply exchanging the recognition element conjugated to the nucleic acid strands, we demonstrated the detection of six different antibodies (anti-Dig, anti-DNP, anti-HIV, anti-MUC, Cetuximab and a bispecific antibody). A direct comparison with a commercial ELISA kit for the detection of Cetuximab demonstrates that MAIGRET is highly competitive in terms of sensitivity and simplicity of reaction steps. Using a competitive format, MAIGRET can also support the detection of antigens (EGFR, Muc1 and p17 peptide).

Compared to a recently reported CRISPR-based approach for protein detection (i.e. UCAD) [22], MAIGRET is slightly less sensitive (fM detection limit for MAIGRET compared to aM detection limit for UCAD). Despite this limitation, MAIGRET could in principle offer more control over the final signal (i.e. less leakage) as it is based on a cell-free transcription reaction rather than DNA polymerization. MAIGRET also appears to be more versatile and can be customized to different targets, for example a bispecific antibody. The antibody-guided CRISPR-based approach proposed here could also have applications beyond the fields of sensing and diagnostics. For example, the ability to direct CRISPR-based cleavage reactions in response to the binding of a target antibody can be used as a checkpoint in CRISPR-based clinical systems, for example by designing genetic circuits that can generate target-responsive gene-based therapies.

## Supplementary Material

gkaf238_Supplemental_File

## Data Availability

All data are available in the manuscript or in the supplementary information. Materials that support the findings of this study are available from the corresponding author upon request.

## Supplementary data

Supplementary data is available at NAR online.
