# Supplementary material for: MAIGRET: a CRISPR-based immunoassay that employs antibody-induced cell-free transcription of CRISPR guide RNA strands
Source: Nucleic Acids Res. 2025 Mar 28;53(6):gkaf238. doi: 10.1093/nar/gkaf238 (PMC11952961; doi:10.1093/nar/gkaf238)
Supplement: gkaf238_Supplemental_File [file gkaf238_supplemental_file.docx]

**MAIGRET: a CRISPR-based immunoassay that employs antibody-induced cell-free transcription of CRISPR guide RNA strands**

Francesca Miceli^1^, Sara Bracaglia^1^, Daniela Sorrentino^1,2^, Alessandro Porchetta^1,3^, Simona Ranallo^1,3*^, Francesco Ricci^1,3*^

^1^ Department of Chemistry, University of Rome, Tor Vergata, Via della Ricerca Scientifica, 00133, Rome, Italy

2 Department of Mechanical and Aerospace Engineering and of Bioengineering, University of California at Los Angeles, 420 Westwood Plaza, Los Angeles, California 90095, United States

^3^ Istituto Nazionale Biostrutture e Biosistemi, INBB, Via dei Carpegna, 00165 Rome, Italy

* To whom correspondence should be addressed. Tel. +39 06 72594422; Email. [francesco.ricci@uniroma2.it](mailto:francesco.ricci@uniroma2.it); [simona.ranallo@uniroma2.it](mailto:simona.ranallo@uniroma2.it)

**Table S1: MAIGRET for anti-Dig antibody detection**

| **Name** | **Sequences** |
| --- | --- |
| Non template strand | 5’- TGA GGA ATA CAT ATA C**TA ATA** CGA CTC ACT ATA GGG TAA TTT CTA CTA AGT GTA GAT GTC TAC ACA TGG CTA AAT CT -3’ |
| Incomplete template strand | 5’- AGA TTT AGC CAT GTG TAG ACA TCT ACA CTT AGT AGA AAT TA C CC T ATA GTG AGT CG - 3’ |
| Synthetic crRNA | 5’- GGG UAA UUU CUA CUA AGU GUA GAU GUC UAC ACA UGG CUA AAU CU - 3’ |
| Non-Target strand | 5’ - TTT AGT CTA CAC ATG GCT AAA TCT - 3’ |
| Target strand | 5’- AGA TTT AGC CAT GTG TAG ACT AAA -3’ |
| Cas12 Reporter | 5’- (6-Fam) - CTC TC A TTT TTA GAG AG - (BHQ1) - 3’ |
| Split input#1_Stem16 | 5’- (Dig) - TTT TTT TTT TTT – *TGC A CTG GTC CAC TCT* - TT - G TAT TCC TCA - 3’ |
| Split input#1_Stem12 | 5’- (Dig) - TTT TTT TTT TTT - *CTG GTC CAC TCT* - TT - G TAT TCC TCA - 3’ |
| Split input#2_Stem16 | 5’ - **TAT TA**G TAT AT – TT – *AGA GTG GAC CAG TGC A* - TTT TTT TTT TTT - (Dig) -3’ |
| Split input#2_Stem14 | 5’ - **TAT TA**G TAT AT – TT – *AGA GTG GAC CAG TG* - TTT TTT TTT TTT - (Dig) - 3’ |
| Split input#2_Stem12 | 5’ - **TAT TA**G TAT AT – TT – *AGA GTG GAC CAG* - TTT TTT TTT TTT - (Dig) - 3’ |
| Split input#2_Stem10 | 5’ - **TAT TA**G TAT AT – TT – *AGA GTG GAC C* - TTT TTT TTT TTT - (Dig) - 3’ |
| Split input#2_Stem8 | 5’ - **TAT TA**G TAT AT – TT – *AGA GTG GA* – TTT TTT TTT TTT - (Dig) - 3’ |
| Split input#2_Stem6 | 5’ - **TAT TA**G TAT AT – TT – *AGA GTG* – TTT TTT TTT TTT - (Dig) - 3’ |
| Split input#2_Stem4 | 5’ - **TAT TA**G TAT AT – TT – *AGA G* – ATT TTT TTT TTT - (Dig) - 3’ |
| Split input#2_Stem2 | 5’ - **TAT TA**G TAT AT – TT – *AG* – TTT TTT TTT TTT - (Dig) – 3’ |
| Split input#2_Stem0 | 5’ - **TAT TA**G TAT AT – TT – *TGT GTT* - TTT TTT TTT TTT - (Dig) – 3’ |
| Linear Input control | 5’- TAT TAG TAT ATG TAT TCC TCA - 3’ |
| Stem-loop control | 5’- **TAT TA**G TAT AT -TT – GTC TGC AG - TTT TTT TTT - CT- GCA GAC- TT -G TAT TCC TCA - 3’ |

In the above sequences and those reported below with other templates the sequence denoted in bold and underlined indicates the complementary portions between the single-stranded non-template strand and the split input strands (split input #1 and split input#2). The sequence denoted in *italics* indicates the elements forming the stem.

In the above antigen-conjugated DNA strands Dig was conjugated using EDC/NHS to an amine attached via a 5-carbon linker on either the 5’- or 3’-end of the DNA.

For the experiments shown in Figure 2B-E where we tested different stem lengths we have used the following combinations of split input #1 and split input #2 strands:

| Stem length | Split input #1 stem length | Split input #2 stem length |
| --- | --- | --- |
| 0 | 12 | 0 |
| 2 | 12 | 2 |
| 4 | 12 | 4 |
| 6 | 12 | 6 |
| 8 | 12 | 8 |
| 10 | 12 | 10 |
| 12 | 12 | 12 |
| 14 | 16 | 14 |
| 16 | 16 | 16 |

**Table S2: MAIGRET for anti-DNP antibody detection**

| **Name** | **Sequences** |
| --- | --- |
| Non template strand | 5’- GCA AAT GAC AGT GAC G**TA ATA** CGA CTC ACT ATA GGG AT T TA GAC TAC CCC AAA AAC GAA GGG GAC TAA AAC TCA GTT TTG CAT GGA TTT GCA CA - 3’ |
| Incomplete Template strand | 5’- TGT GCA AAT CCA TGC AAA ACT GA G TTT TAG TCC CCT TCG TTT TTG GGG TAG TCT AAA TC CC TA TAG TGA GTC G - 3’ |
| Synthetic crRNA | 5’- GAU UUA GAC UAC CCC AAA AAC GAA GGG GAC UAA AAC - UCA GUU UUG CAU GGA UUU GCA CA - 3’ |
| Target Strand | 5’- UGU GCA AAU CCA UGC AAA ACU GA - 3’ |
| Cas13 Reporter | 5’- (Cy-3) - CUC UCA UUU UUA GAG AG - (BHQ2) - 3’ |
| Split input#1_Stem12 | 5’- (DNP) - TTT TTT TTT TTT – *GT GCT CAG TTC C* - TT - T GTC ATT TGC - 3’ |
| Split input #2_Stem12 | 5’ - **TAT TA**C GTC AC – TT – *GGA ACT GAG CAC* – TTT TTT TTT TTT - (DNP) - 3’ |
| Split input#2_Stem10 | 5’ - **TAT TA**C GTC AC – TT – *GGA ACT GAG C* – TTT TTT TTT TTT - (DNP) - 3’ |
| Split input#2_Stem8 | 5’ - **TAT TA**C GTC AC – TT – *GGA ACT GA* – TTT TTT TTT TTT - (DNP) - 3’ |
| Split input#2_Stem6 | 5’ - **TAT TA**C GTC AC – TT – *GGA ACT* – TTT TTT TTT TTT - (DNP) - 3’ |
| Split input#2_Stem4 | 5’ - **TAT TA**C GTC AC – TT – *GGA A* – TTT TTT TTT TTT - (DNP) - 3’ |

In the above sequences and those reported below with other templates the sequence denoted in bold and underlined indicates the complementary portions between the single-stranded non-template strand and the split input strands (split input #1 and split input#2). The sequence denoted in *italics* indicates the elements forming the stem.

In these antigen-conjugated strands DNP was attached via a triethylene glycol (TEG) spacer arm on either the 5’- or the 3’- terminus of the DNA.

**Table S3: MAIGRET for anti-HIV antibody detection**

| **Name** | **Sequences** |
| --- | --- |
| Split input#1_Stem12 | 3’ - GTC ACC GCA AAA TAA GA - 5’- 5’ - TT - *CTG GTG CAT CGT* - TT - G TAT TCC TCA - 3’ |
| Split input#2_Stem8 | 5’ - **TAT TA**G TAT AT - TT - *ACG ATG CA* - TT - AGA ATA AAA CGC CAC TG -3’ |
| Peptide-PNA strand | N_term_ – (*ELDRWEKIRLRP*) –CAG TGG CGT TTT ATT CT - C_term_ |

The sequence in parentheses represent the peptide sequences that are terminally conjugated to PNA. In this case we used the same sequences reported in Table S1 for the non-template strand, incomplete template strand, target strand, etc.

**Table S4: MAIGRET for Cetuximab detection**

| **Name** | **Sequences** |
| --- | --- |
| Split input#1_Stem12 | 3’ - GTC ACC GCA AAA TAA GA - 5’- 5’ - TT - *CTG GTG CAT CGT* - TT - G TAT TCC TCA - 3’ |
| Split input#2_Stem8 | 5' - **TAT TA**G TAT AT - TT- *ACG ATG CA -* TTT TTT TT TTT TTT TTT –(EGFR) -3' |
| Antigen-coniugated DNA strand | 5’ - (EGFR) – TTTTTTTTTT CAG TGG CGT TTT ATT CT –3’ |

In this case we used the same sequences reported in Table S1 for the non-template strand, incomplete template strand, target strand, etc.

**Table S5: MAIGRET for anti-MUC antibody detection**

| **Name** | **Sequences** |
| --- | --- |
| Split input#1_Stem12 | 5’ - GGA TAG TCG AAT TTA GT – TT - *CTG GTG CAT CGT -* TT - G TAT TCC TCA – 3’ |
| Split input#2_Stem8 | 5’ - **TAT TA**G TAT AT – TT - *ACG ATG CA* - TT- 3’ 3' TG ATT TAA GCT GAT AGG 5' |
| Peptide-PNA strand | N_term_-ACT AAA TTC GAC TAT CC- (APDTRPAPGSTAPPA) -C_term_ |

The sequence in parentheses represent the peptide sequences that are terminally conjugated to PNA. In this case we used the same sequences reported in Table S1 for the non-template strand, incomplete template strand, target strand, etc.

**Table S6: MAIGRET for Bispecific antibody detection**

| **Name** | **Sequences** |
| --- | --- |
| Split input#1_Stem12 | 5’ - GGA TAG TCG AAT TTA GT -TT- *CTG GTG CAT CGT* - TT G TAT TCC TCA – 3’ |
| Split input#2_Stem8 | 5' - **TAT TA**G TAT AT - TT- *ACG ATG CA* TTT TTT TT TTT T-TT TTT –(EGFR) -3' |
| Peptide-PNA strand | N_term_-ACT AAA TTC GAC TAT CC- (APDTRPAPGSTAPPA) -C_term_ |

The sequence in parentheses represent the peptide sequences that are terminally conjugated to PNA. In this case we used the same sequences reported in Table S1 for the non-template strand, incomplete template strand, target strand, etc.


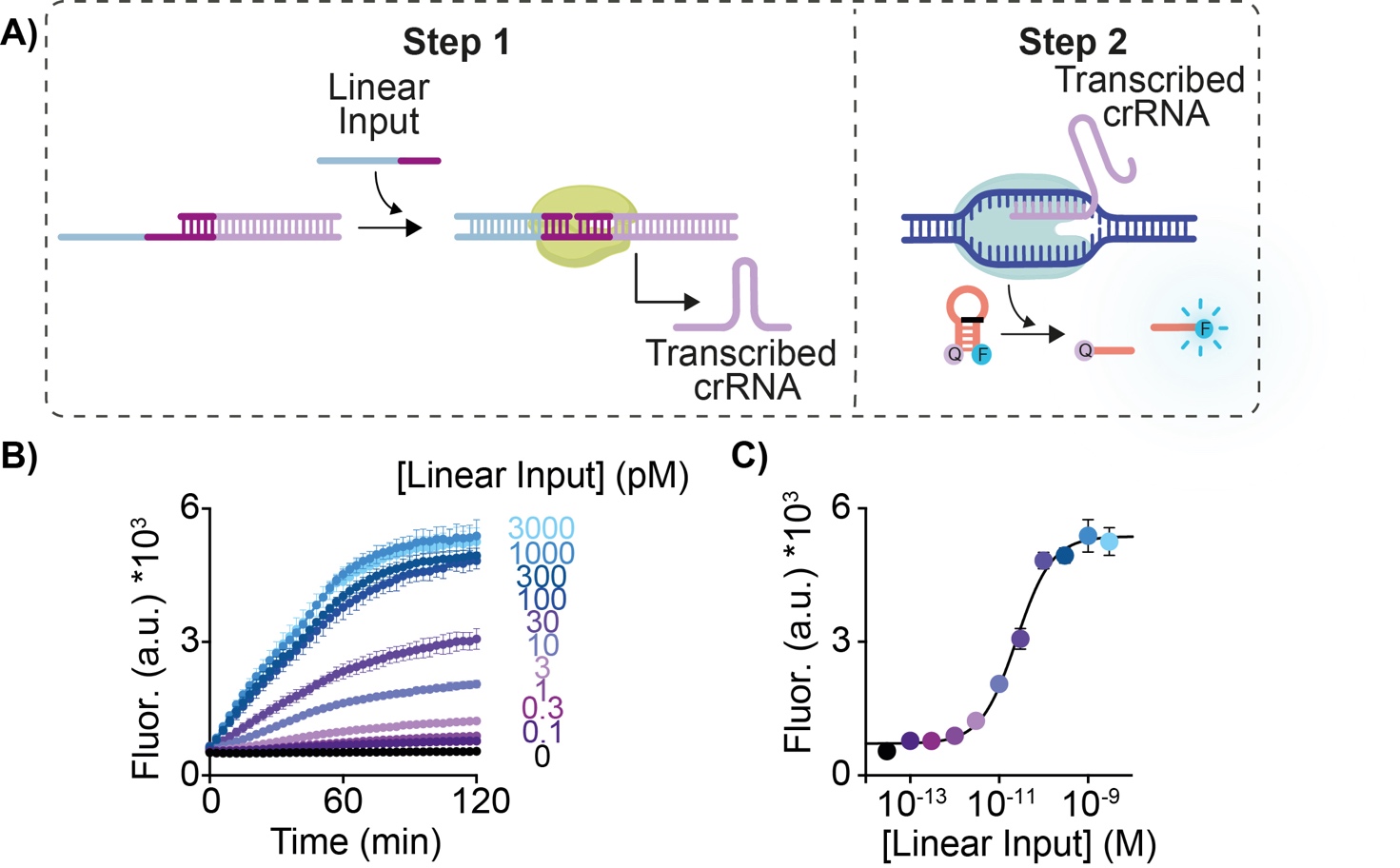


**Figure S1.** (A) Characterization of MAIGRET using a linear control DNA strand to activate the in-vitro transcription of the crRNA. (B) Fluorescence signals and binding curve (C) obtained at increasing concentrations of the linear control strand. The experimental procedure used here is the same described in Figure 2 with the template-activating strand at a concentration of 3 nM.


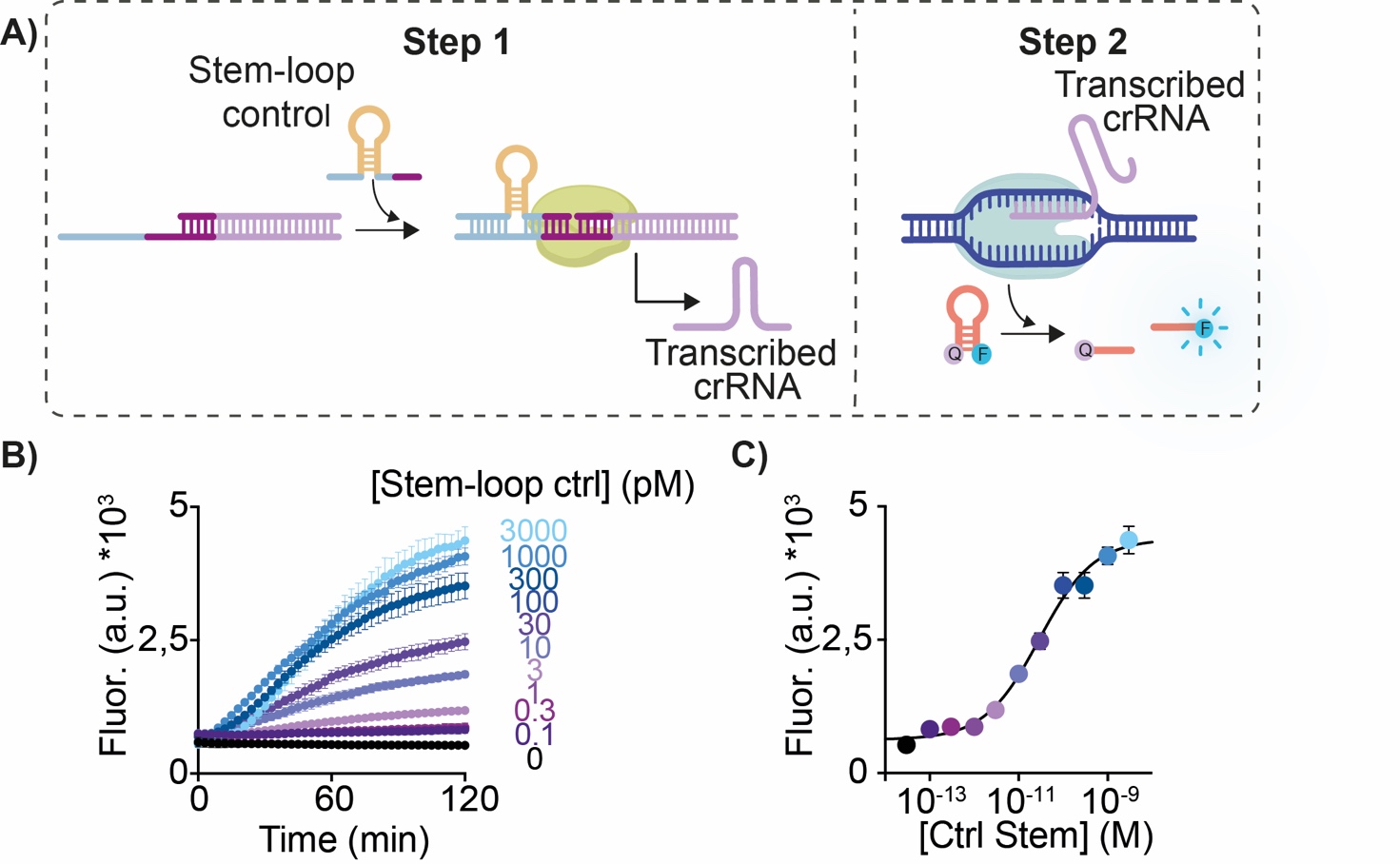


**Figure S2.** (A) Characterization of MAIGRET using a bulge 8-nt stem control DNA strand to activate the in-vitro transcription of the crRNA. (B) Fluorescence signals and binding curve (C) obtained at increasing concentrations of the stem-loop control input strand. The experimental procedure used here is the same described in Figure 2 with the template-activating strand at a concentration of 3 nM.


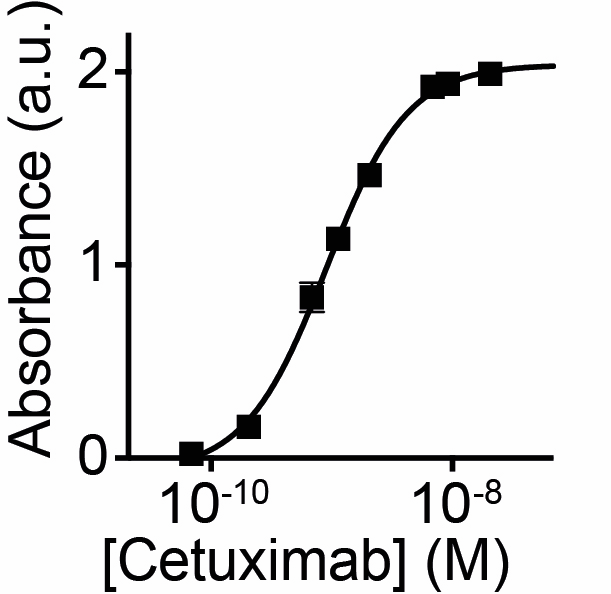


**Figure S3.** Dose-response curve obtained with the ELISA kit for the detection of Cetuximab. The ELISA kit was performed according to the manufacturer’s instructions (see Materials and Methods for details).


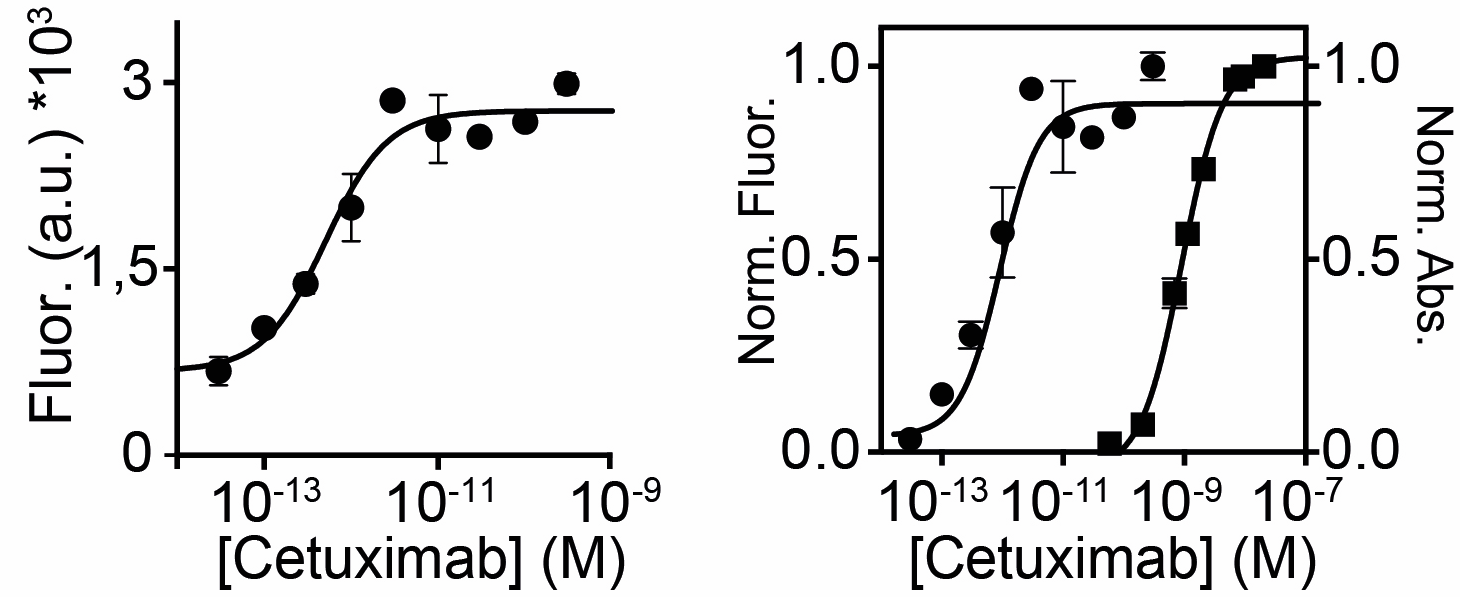


**Figure S4.** Direct comparison (with normalized y-axis scales) between MAIGRET and a commercial ELISA kit for the detection of Cetuximab. Dose-response curves obtained with MAIGRET assay (circles) and the ELISA kit (squares). Experimental details of MAIGRET assay can be found in the legend of Figure 3. The ELISA kit was performed according to the manufacturer’s instructions (see Materials and Methods for details).


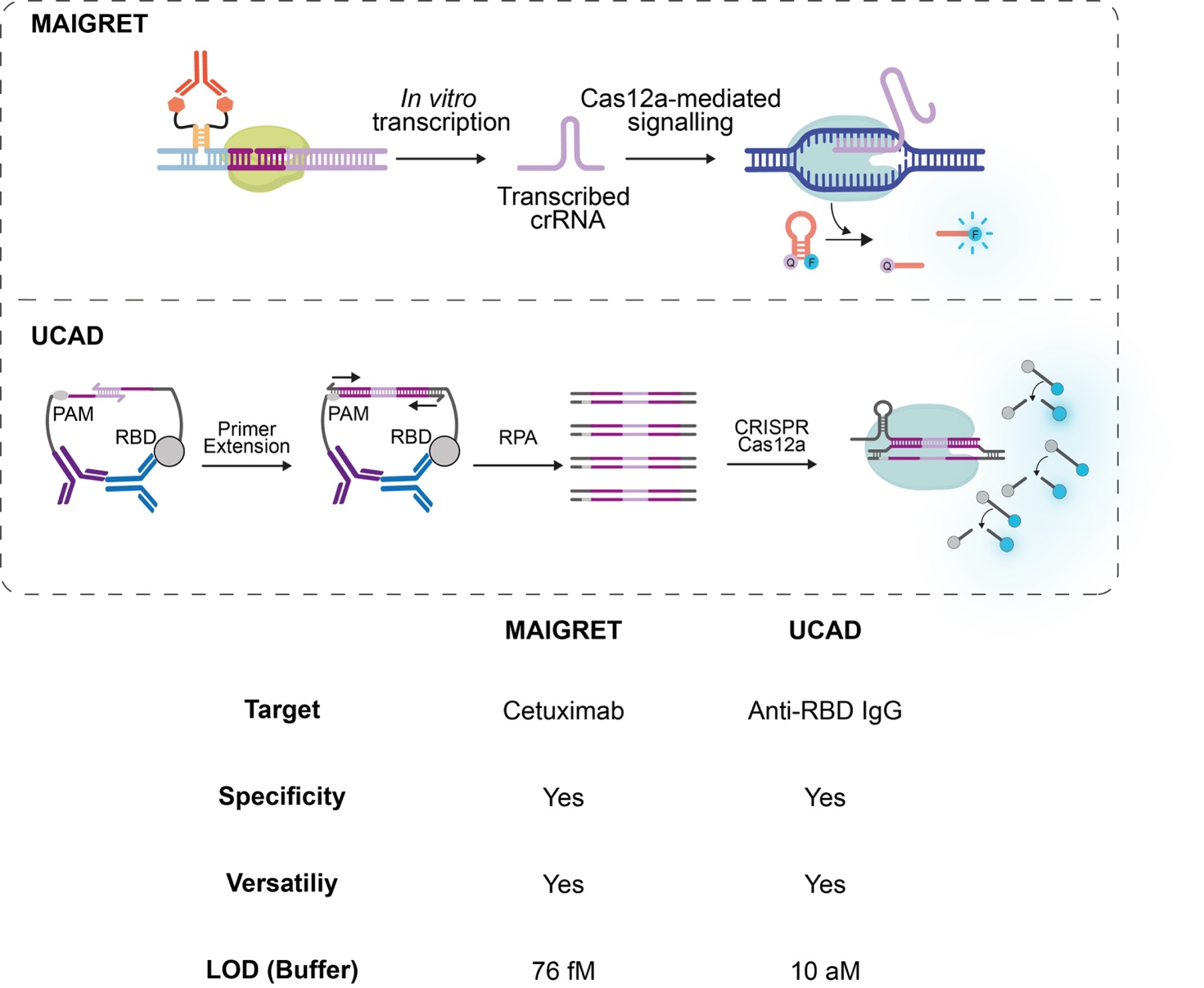


**Figure S5:** Comparison between MAIGRET and UCAD^1^. MAIGRET consist in two steps, including (1) antibody induced transcription reaction of crRNA and (2) the cleavage of a fluorophore-quencher (FQ) labeled ssDNA reporters mediated by CRISPR-Cas12a. UCAD, instead, consists of three steps, including (1) antibody-specific primer extension to produce dsDNA barcodes; (2) RPA amplification; and (3) the cleavage of fluorophore-quencher (FQ) labeled ssDNA reporters mediated by CRISPR-Cas12a. In the table the reported LOD, specificity and versatility (in terms of adaptability to the detection of different targets) has also been reported.

**
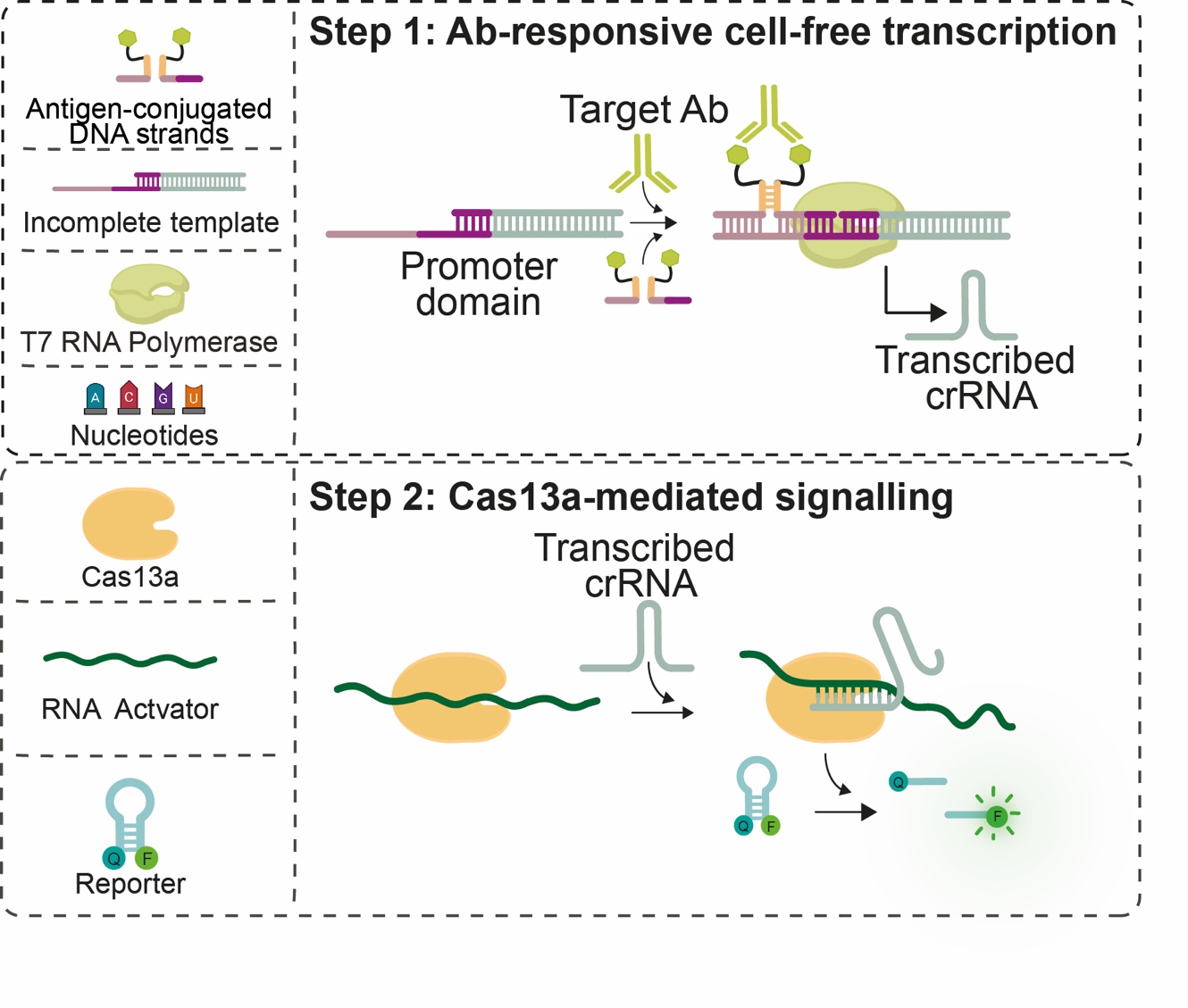
**

**Figure S6:** MAIGRET employing Cas13a RNA collateral activity in step 2 for the detection of Anti-DNP antibodies. The first step of MAIGRET involves the use of DNP-conjugated DNA strands that can hybridize to an incomplete synthetic DNA template after binding of the antibody and induce cell-free transcription of a CRISPR RNA (crRNA) guide strand by T7 RNA polymerase. In the second step, the transcribed crRNA triggers the collateral cleavage of a fluorescent RNA hairpin reporter.

**
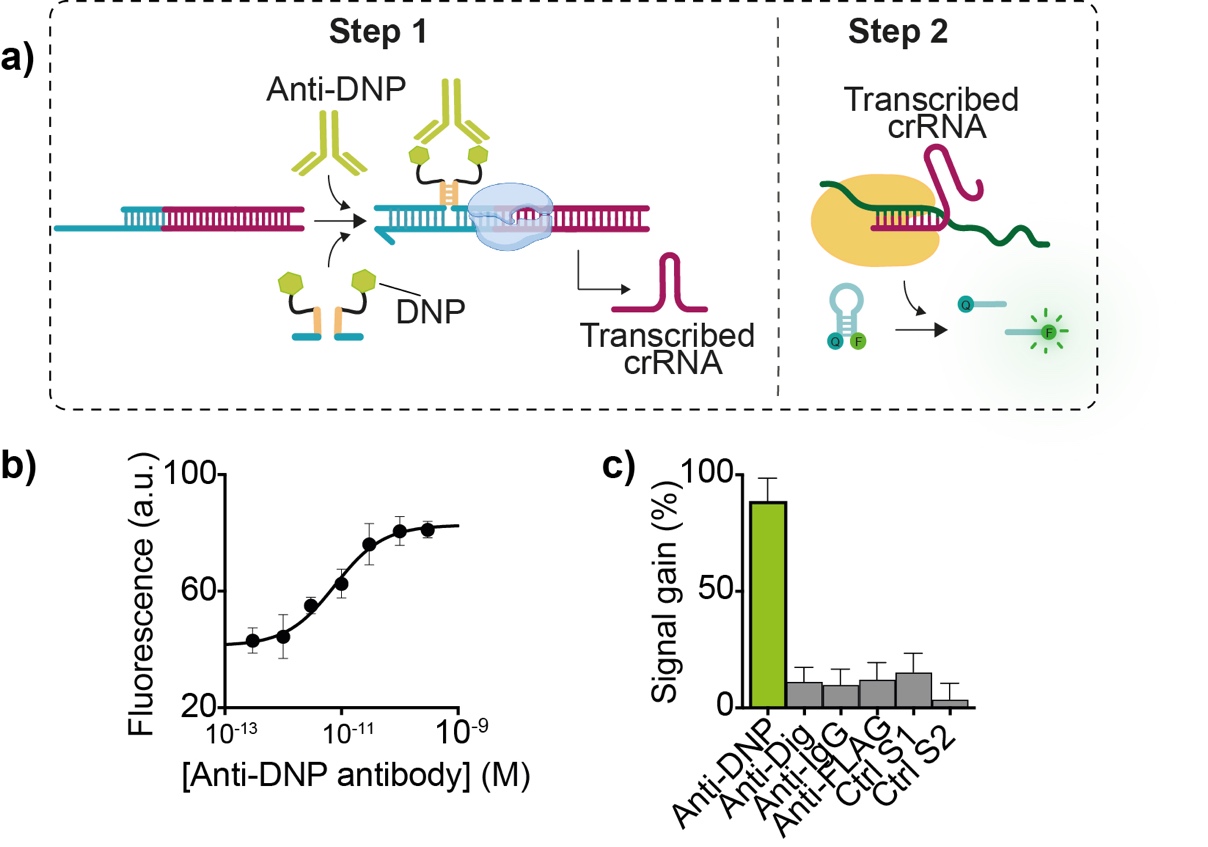
**

**Figure S7:** (a) Simplified scheme of MAIGRET for the detection of anti-DNP antibodies using DNP-conjugated DNA strands and Cas13a enzyme. (b) Binding curve at increasing anti-DNP concentrations using the DNP-conjugated DNA strands with 8-nt complementary moiety. (c) Fluorescence signals obtained in the presence of saturating concentrations (300 pM) of anti-DNP antibody and non-specific antibodies. The fluorescence signals from control experiments (Ctrl S1 and Ctrl S2) performed in the presence of only one of the Dig-conjugated DNA strands are also shown here. The experiments were conducted at 37 °C in a 10 μL solution containing 50% diluted bovine serum, in presence of T7-RNAP (10 U/mL) and the required nucleotides (each at 10 mM) supplemented with inactive template (300 pM), the antigen-conjugated strands (each at 3 nM) and the Anti-DNP antibody as indicated. The transcription reaction was allowed to proceed for 120 min and then an aliquot was transferred to 18 μL of 80 mM HEPES, 200 mM KCl, 20 mM MgCl_2_, 0.4 mg/mL BSA, pH 7.5 solution containing Cas13a (3 nM), the RNA activator (200 pM), and an RNA reporter (30 nM) and the fluorescence signal measured at 595 nm. The values represent averages of three separate measurements and the error bars reflect the standard deviations.


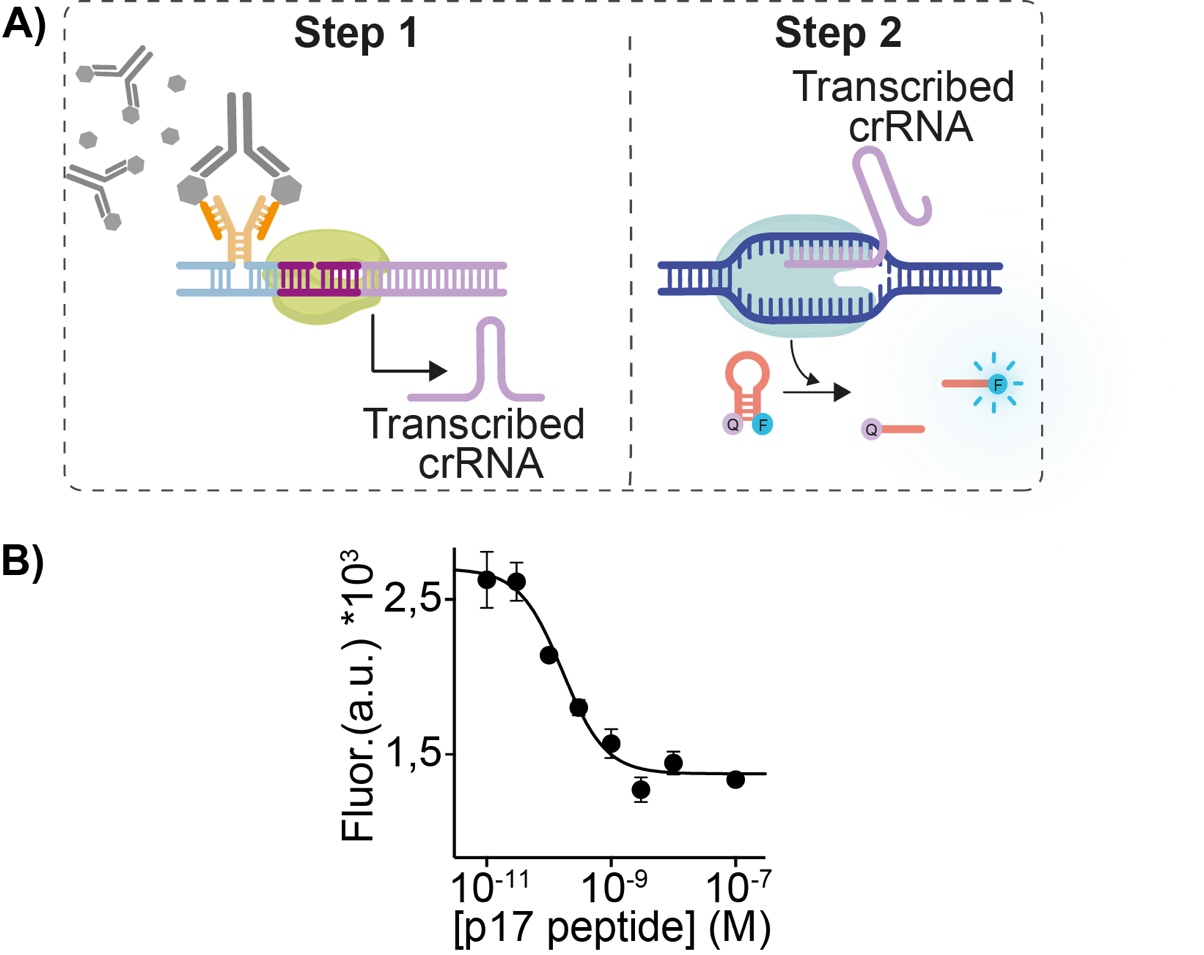


**Figure S8.** (A) General scheme of the competitive format of MAIGRET for the detection of p17 peptide. (B) Binding curves at increasing p17 peptide using the modular version of MAIGRET described in Figure 3. The experiments here were performed as described in Figures 3 with the exception that the solution of the transcription step (step 1) of the assay was supplemented with a fixed concentration (3 nM) of Anti-HIV antibody.

**REFERENCES**

1. Tang,Y., Song,T., Gao,L., Yin,S., Ma,M., Tan,Y., Wu,L., Yang,Y., Wang,Y., Lin,T. and Li, F. (2022) A CRISPR-based ultrasensitive assay detects attomolar concentrations of SARS-CoV-2 antibodies in clinical samples. *Nat. Comm.*, **13**, 4667.
